# The use of DNA barcoding to monitor the marine mammal biodiversity along the French Atlantic coast

**DOI:** 10.3897/zookeys.365.5873

**Published:** 2013-12-30

**Authors:** Eric Alfonsi, Eleonore Méheust, Sandra Fuchs, François-Gilles Carpentier, Yann Quillivic, Amélia Viricel, Sami Hassani, Jean-Luc Jung

**Affiliations:** 1 Laboratoire BioGeMME (Biologie et Génétique des Mammifères Marins dans leur Environnement), Université Européenne de Bretagne & Université de Bretagne Occidentale, UFR Sciences et Techniques, 6 Av. Victor Le Gorgeu - CS93837 - 29238 Brest Cedex 3, France; 2 Laboratoire d’Etude des Mammifères Marins (LEMM), Océanopolis, port de plaisance, BP 91039, 29210 Brest Cedex 1, France; 3 Observatoire PELAGIS, UMS 3462, CNRS-Université de La Rochelle, Pôle analytique, 5 allée de l’océan, 17000 La Rochelle, France; 4 Littoral, Environnement et Sociétés, UMR 7266, CNRS-Université de La Rochelle, 2 rue Olympe de Gouges, 17000 La Rochelle, France

**Keywords:** DNA barcoding, COI, control region, marine mammals, cetaceans, pinnipeds, biodiversity monitoring, stranding network

## Abstract

In the last ten years, 14 species of cetaceans and five species of pinnipeds stranded along the Atlantic coast of Brittany in the North West of France. All species included, an average of 150 animals strand each year in this area. Based on reports from the stranding network operating along this coast, the most common stranding events comprise six cetacean species (*Delphinus delphis*, *Tursiops truncatus*, *Stenella coeruleoalba*, *Globicephala melas*, *Grampus griseus*, *Phocoena phocoena*)and one pinniped species (*Halichoerus grypus*). Rare stranding events include deep-diving or exotic species, such as arctic seals. In this study, our aim was to determine the potential contribution of DNA barcoding to the monitoring of marine mammal biodiversity as performed by the stranding network.

We sequenced more than 500 bp of the 5’ end of the mitochondrial COI gene of 89 animals of 15 different species (12 cetaceans, and three pinnipeds). Except for members of the Delphininae, all species were unambiguously discriminated on the basis of their COI sequences. We then applied DNA barcoding to identify some “undetermined” samples. With again the exception of the Delphininae, this was successful using the BOLD identification engine. For samples of the Delphininae, we sequenced a portion of the mitochondrial control region (MCR), and using a non-metric multidimentional scaling plot and posterior probability calculations we were able to determine putatively each species. We then showed, in the case of the harbour porpoise, that COI polymorphisms, although being lower than MCR ones, could also be used to assess intraspecific variability. All these results show that the use of DNA barcoding in conjunction with a stranding network could clearly increase the accuracy of the monitoring of marine mammal biodiversity.

## Introduction

The aim of DNA barcoding is to concentrate the efforts of molecular taxonomists on a single part of the mitochondrial genome, chosen because it presents portions conserved across taxa that are appropriate for primer design, while including polymorphism among and within species (Hebert et al. 2003, 2004). This DNA sequence, targeted as the 5’ end of the gene coding for the subunit 1 of the cytochrome *c* oxidase subunit I (COI), is sufficiently diverse so as to allow the specific identification of a great majority of animal species. Numerous studies have proven the success of this approach in the animal kingdom, and using various sources of tissue samples (e.g. Lambert 2005, Clare et al. 2007, Dawnay et al. 2007, Hajibabaei et al. 2007, Borisenko et al. 2008, Ward et al. 2009, Shokralla et al. 2010). Today (June 2013), a database, accessible at http://www.boldsystems.org, groups DNA barcode sequence data for more than 133,000 animal species, and offers a powerful identification tool for new specimens (Ratnasingham and Hebert 2007).

DNA barcoding also possesses some inherent limitations (Valentini et al. 2009): it is based on a single locus on the mitochondrial genome so that it is only maternally inherited (Hartl and Clark 2007), it can show heteroplasmy (Kmiec et al. 2006, Vollmer et al. 2011) or may exist as nuclear copies. Some of these limitations have been well-exposed (Ballard and Whitlock 2004, Toews and Brelsford 2012). The use of DNA barcoding for species delimitation also requires that interspecific divergence is higher than the intraspecific divergence. Although this has been shown to be true in numerous taxonomic groups, opposite examples also exist (Amaral et al. 2007, Wiemers and Fiedler 2007, Viricel and Rosel 2012).

In the present study, we assess the contributions that DNA barcoding could provide to the monitoring of the marine mammal biodiversity along the coasts of Brittany, in the North West of France. For almost 20 years, the stranding network has been collecting data and, when possible, sampling, each time a marine mammal stranding is reported. Field correspondents are organized in a geographical area covering the entire Brittany coasts. The network is coordinated regionally by Océanopolis (Brest, France), and nationally by *Pelagis* (La Rochelle, France).

DNA barcoding could be useful for the monitoring of marine mammal strandings at different levels. First, by confirming the quality and the reproducibility of a species identification made by the field correspondents. Beside common species, which are often encountered and easily identified, exotic or deep living species represent rare stranding events. In such cases, DNA barcoding could provide a confirmation or an additional degree of precision of taxonomic determination (Thompson et al. 2012). Second, DNA barcoding can help specifying species identifications in those cases where the taxonomic identification was made only to the genus or family levels. This is often due to incomplete or highly degraded carcasses. DNA barcoding also is a valuable and cost effective alternative to the taking of the head or skull of the animals. Third, genetic data collected for DNA barcoding generally include intraspecific variation, which allows downstream population-level analyses including the detection of genetic structure and, in some cases, monitoring population movements. A long-term use of the barcoding approach would therefore clearly increase the significance and the precision of marine mammal stranding monitoring. Migration or movement of populations or groups of a particular species can be highlighted, thus revealing e.g. environmental changes leading to these movements (Pauls et al. 2012).

We evaluated the usefulness of DNA barcoding in the monitoring of marine mammal biodiversity along the coasts of Brittany at three levels: by confirming the taxonomic identification performed by field correspondents, by identifying degraded carcasses or parts of carcasses, and by determining intraspecific variations for two species commonly found off Brittany, the harbour porpoise and the grey seal. For this last part of our study, we also compared COI and the mitochondrial control region in terms of their effectiveness in species identification.

## Methods

### Collection of data and samples

The CRMM (Centre de Recherche sur les Mammifères Marins, La Rochelle, France), presently the Joint Service Unit PELAGIS, UMS 3462, University of La Rochelle-CNRS has created the French marine mammal stranding recording program at the beginning of the 70s. The network comprises about 260 field correspondents, members of organizations or volunteers (Peltier et al. 2013).

Since 1995, the LEMM (Laboratoire d’Etude des Mammifères Marins, Océanopolis, Brest, France) has coordinated this network at a regional scale in Brittany, North West of France. Data are collected from the Brittany coastlines, analyzed, and then added to the central database maintained in La Rochelle. The Brittany coasts have been divided into 18 sections covering the whole coastline (Jung et al. 2009). In each of these areas, correspondents are trained in the analysis of stranded marine mammals. Taxonomic identification and characteristic measurements are performed following a standard procedure. The LEMM therefore compiles standardized data on a large proportion of cetaceans stranded on the Brittany coasts on a yearly basis. Whenever possible, skin, blubber, muscle and teeth samples are also collected in the field from each stranded animal. Samples are then kept in absolute ethanol or dry at -20 °C until analyses. Some harbour porpoise samples, described in the Appendix 1 and in Alfonsi et al. (2012), were stranded or by-caught in the Bay of Biscay (Atlantic coast of France).

### Genomic DNA extraction, amplification and sequencing of COI and MCR (mitochondrial control region)

Genomic DNA was extracted from blood samples or from muscle and skin tissues using a standardized protocol and the DNeasy Blood and Tissue kit (Qiagen), following the instructions of the manufacturer. The quality and the concentration of all the DNA extracts were estimated by agarose gel electrophoresis and by spectrophotometry using a Nanodrop 1000 (Thermo Scientific).

A 736 base-pair (bp) fragment of the 5’ region of the COI fragment (position 5352 to 6087 of the complete mitochondrial genome of the harbour porpoise, GenBank acc. no. AJ554063), was amplified using two newly designed primers, LCOIea (5’-tcggccattttacctatgttcata-3’) and HBCUem (5’-ggtggccgaagaatcagaata-3’). The 50 µl PCR final volume included approximately 50 ng of genomic DNA, and 25 pmole of each primer in the Hotgoldstar master mix x 1 (Eurogentec) with a final concentration of MgCl_2_ of 2.5 mM. After an initial denaturation step of 10 min at 95 °C, the thermocycle profile consisted of 32 cycles for cetaceans or 35 cycles for pinnipeds at 95 °C for 30 s, 53 °C for 30 s and 72 °C for 60 s, with a final extension at 72 °C for 10 min.

For some animals, we also amplified and sequenced another part of the mitochondrial genome including the control region (MCR). For cetaceans, the primers and reaction conditions are described in (Alfonsi et al. 2012). For pinnipeds, two newly designed primers LMCRHgem 5’-tcatacccattgccagcattat-3’ and HMCRHgem 5’-taccaaatgcatgacaccacag-3’ amplified a 693 bp fragment from position 16160 to 55 of the *Halichoerus grypus* complete mitochondrial genome sequence (GenBank acc. no. X72004). PCR reaction conditions were the same as described above for pinnipeds, with the hybridization temperature set to 53 °C. PCR products were purified using the “MinElute PCR Purification Kit” and sequenced by a commercial sequence facility (Macrogen, Korea).

Electropherograms were analyzed and edited manually using the Sequence scanner software (Applied Biosystems), and alignments were produced using CLUSTAL W (Thompson et al. 1994) with default settings in Bioedit (Hall 1999). All sequences were analyzed using the Barcode of Life Data Systems (BOLD) interface (accessible at http://www.boldsystems.org), and were also compared to GenBank data using BLAST (Benson et al. 2010).

DNA sequences and specimen information have been added to two BOLD projects. The first project includes specimens for which the species had been identified without doubt using classical morphological identification, and is referred to as IMMB (Identified Marine Mammals in Brittany). The IMMB project is a part of the campaign “barcoding mammals of the world”. The second project, UMMB (Unidentified Marine Mammals in Brittany), includes specimens only identified to the genus or to higher taxonomic levels. This second project is a part of the campaign “barcoding application”.

Genetic distances (intraspecific, interspecific and minimal distance to the nearest neighbour) were calculated using the Kimura 2-parameter (K2P) model (Kimura 1980) and the MUSCLE alignment algorithm on the BOLD user interface or using the software MEGA5 (Tamura et al. 2011). Neighbour-Joining trees based on the K2P-model were built using the BOLD user interface. DnaSP v5.10 was used to calculate haplotype and nucleotide diversities (Librado and Rozas 2009). We used non-metric multidimensional scaling (nMDS) to represent MCR distances graphically and to discriminate closely related species within the *Stenella*-*Tursiops*-*Delphinus* complex (LeDuc et al. 1999, McGowen 2011, Perrin et al. 2013). Distance matrices were computed with the K2P-model using DNAdist (Felsenstein 1989) and were then analyzed by nMDS using Statistica (Statsoft 2005). Posterior probabilities were calculated by a LDA (linear discriminant analysis) on coordinates given by the nMDS. Phylogenetic relationships among COI sequences of harbour porpoise were depicted using a median joining network of haplotypes using Network v4.6 (www.fluxus-engineering.com).

## Results

**Figure 1.**
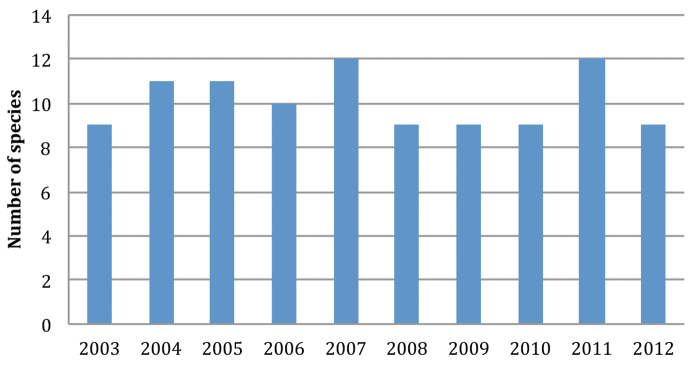
Numbers of different species of marine mammals stranded along the coasts of Brittany (North West of France) in the period 2003–2012.

From 2003 to 2012, 1530 marine mammal strandings were recorded along the coastline of Brittany (Table 1). Fourteen species of cetaceans and five species of pinnipeds were identified. The most frequent cetaceans were six indigenous species of the Brittany waters, viz. five members of the Delphinidae (*Delphinus delphis*, *Tursiops truncatus*, *Stenella coeruleoalba*, *Globicephala melas*, *Grampus griseus*), and the harbour porpoise (*Phocoena phocoena*). Two members of the Zyphiidae (*Hyperoodon ampullatus* and *Ziphius cavirostris*), three other species of Delphinidae (*Lagenorhynchus acutus*, *Orcinus orca* and *Stenella frontalis*), one species of Physeteridae (*Physeter macrocephalus*) and two mysticete species (*Balaenoptera acutorostrata* and *Balaenoptera physalus*) were rare stranding events. *Halichoerus grypus* was by far the most commonly encountered pinniped, far before *Phoca vitulina*, and some uncommon arctic seals (*Phoca hispida*, *Cystophora cristata* and *Phoca groenlandica*). Between 9 and 12 different marine mammal species stranded each year (Figure 1).

**Table 1.** Strandings of marine mammals along the coasts of Brittany, North West of France (2003–2012)

	2003	2004	2005	2006	2007	2008	2009	2010	2011	2012	Total
**Cetaceans**											
*Balaenoptera acutorostrata*								1	1		2
*Balaenoptera physalus*	1	2			2	3			4	2	14
Delphinidae (undetermined)	40	30	36	22	15	9	9	6	16	8	191
*Delphinus delphis*	56	61	109	53	51	56	40	39	72	57	594
*Globicephala melas*	6	5	7	1	1	2	1	2	1	2	28
*Grampus griseus*	2	1	7	3	1	7	2	1	2	4	30
*Hyperoodon ampullatus*									1		1
*Lagenorhynchus acutus*				1	2				1	1	5
*Orcinus orca*		1									1
*Phocoena phocoena*	18	13	12	15	20	23	9	10	15	11	146
*Physeter macrocephalus*		2			1						3
*Stenella coeruleoalba*	1		7	9	8	4	5	9	6	3	52
*Stenella frontalis*			1								1
*Tursiops truncatus*	6	2	7	6	4	5	3	8	3	3	47
*Ziphius cavirostris*					1		1				2
Mysticeti (undetermined)			1	4							5
Odontoceti (undetermined)	5	1	1	3	1						11
Cetacea (undetermined)				3	3	2			1		9
**Pinnipeds**											
*Cystophora cristata*			1	3							4
*Halichoerus grypus*	20	29	41	37	51	41	37	13	34	24	327
*Phoca groenlandica*		1									1
*Phoca vitulina*	1	1	2	1	1	1		2	3		12
*Pusa hispida*			1				1				2
Phocidae (undetermined)	5		7	4	13	4	5		2	1	41
Unknown										1	1
**Total**	**161**	**149**	**240**	**165**	**175**	**157**	**113**	**91**	**162**	**117**	**1530**

Members of the stranding network are trained to identify the stranded animals. Nevertheless, 258 animals (16.8% of the strandings) were not characterized to the species level, generally because of an advanced state of decomposition of the animal body, sometimes in conjunction with bad field-work conditions.

### COI sequencing and analysis from different marine mammal samples

**Figure 2.**
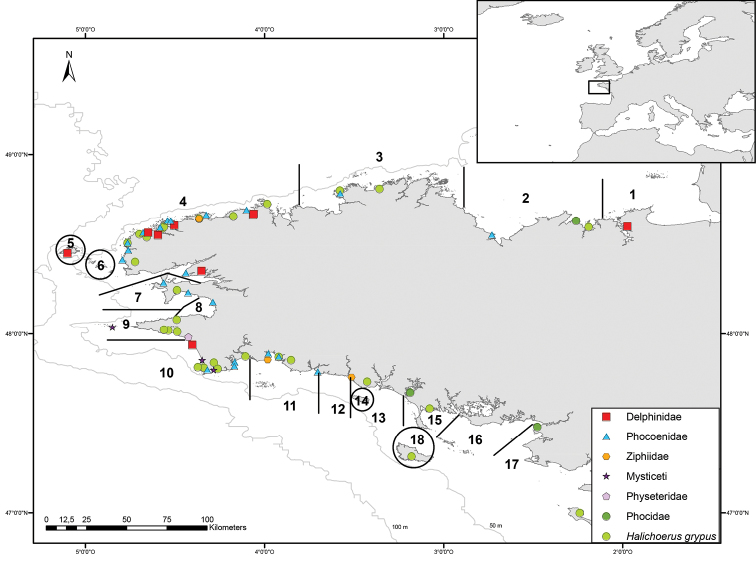
Organization of the stranding network in Brittany (North West of France) and localization of the stranded specimens used in this study. Numbers indicate the 18 geographic sections of the stranding network in this area. The map was drawn using ArcGIS Desktop: Release 9.3.1 (Environmental Systems Research Institute, Redlands, CA, USA) with WGS 84 coordinates.

DNA was extracted from 92 stranded animals, i.e. from dead cetaceans and pinnipeds, but also from 40 grey seals stranded alive, which were treated in the care center of Océanopolis (Brest, France) and from which a small blood sample was taken and kept at -20 °C. All the samples came from animals stranded at the coasts of Brittany, except for one grey seal (Hgc406), which stranded alive in Spain in 2009 and which was transported to the care center (Figure 2). Our sampling included 12 species of cetaceans, and three species of pinnipeds (Table 2). Two species were very common, the harbour porpoise (29 samples) and the grey seal (44 samples), thus allowing intraspecific distance analyses.

**Table 2.** Numbers of samples included in the IMMB project.

Cetaceans (12 species)	
*Balaenoptera acutorostrata*	1
*Balaenoptera physalus*	1
*Delphinus delphis*	1
*Grampus griseus*	3
*Hyperoodon ampullatus*	2
*Lagenorhynchus acutus*	2
*Phocoena phocoena*	29
*Physeter macrocephalus*	1
*Stenella coeruleoalba*	1
*Stenella frontalis*	1
*Tursiops truncatus*	1
*Ziphius cavirostris*	1
**Pinnipeds (3 species)**	
*Cystophora cristata*	2
*Halichoerus grypus*	44
*Phoca vitulina*	2
**Total (15 species)**	92

**Figure 3.**
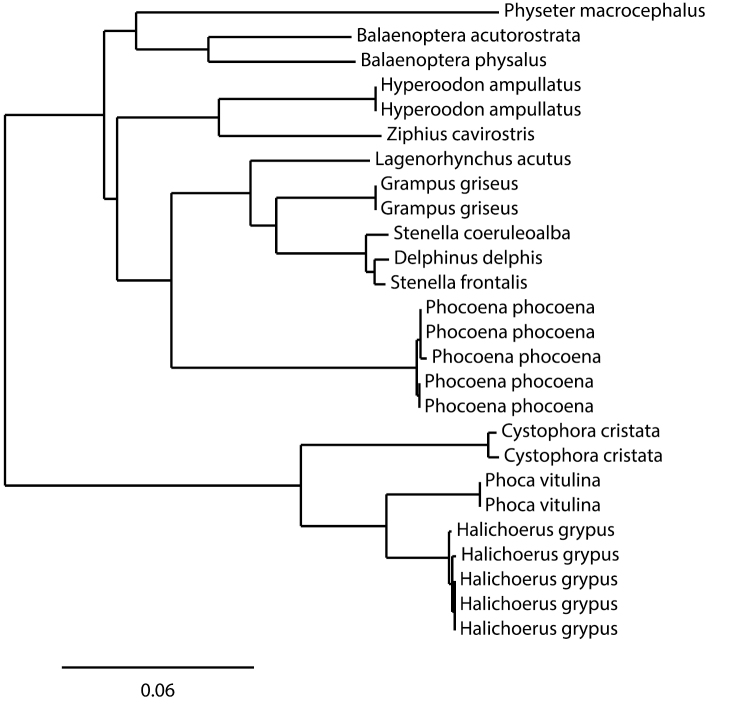
Neighbour-Joining tree of major species of marine mammals, based on K2P-distances calculated from 507 bp of COI. All sequences come from the IMMB project on BOLD, and only 5 harbour porpoise and 5 grey seal samples among those of the IMMB project have been included in the analysis.

A COI amplicon was recovered from 89 samples, and good quality sequences of more than 500 bp were obtained for all samples (GenBank accession numbers KF281608–KF281697). The sequence alignment used in the analyses was 507 bp long. About 32% of the positions were polymorphic in the cetaceans and 13.1% in the pinnipeds (Table 3). The maximal intraspecific distance was 0.46% for the grey seal and 0.83% for the harbour porpoise. The COI sequences of three species of the Delphininae (*Stenella frontalis*, *Stenella coeruleoalba* and *Delphinus delphis*) showed very low interspecific distances (0.84% between *Delphinus delphis* and the nearest species *Stenella frontalis*, and 1.18% between the two *Stenella* species). All other interspecific distances were above 3.9% for pinnipeds and above 6% for cetaceans. The Neighbour-Joining (NJ) tree built on the BOLD interface using K2P-distances (Figure 3) confirms that, except for of the Delphininae, all the cetacean and pinniped species analyzed are distinguished unambiguously.

**Table 3.** Polymorphism levels of COI between 12 species of marine mammals stranded in Brittany, and comparison with intra-species variation for harbour porpoise and grey seal.

	Total	Cetaceans (12 species)	Pinnipeds (3 species)	Harbour porpoises	Grey seals
Number of species	14	11	3	1	1
Number of sequences	89	41	48	45*	44
Length (bp)	507	508	656	610	658
Polymorphic sites	186	163	86	8	7
Polymorphism (%)	36.7	32.1	13.1	1.3	1.06
Minimal distance to NN	-	0.84	3.9	13.46	3.9
Maximal distance to NN	-	17.3	11.2	-	-
Maximal intraspecific distance	-	-	-	0.83	0.46

*This sampling includes 28 harbour porpoises stranded along the coasts of Brittany, and 17 more samples, stranded or by-caught in the Bay of Biscay, included to better characterize intraspecific variation. NN: nearest neighbour.

### Taxonomic identification of undetermined samples

**Figure 4.**
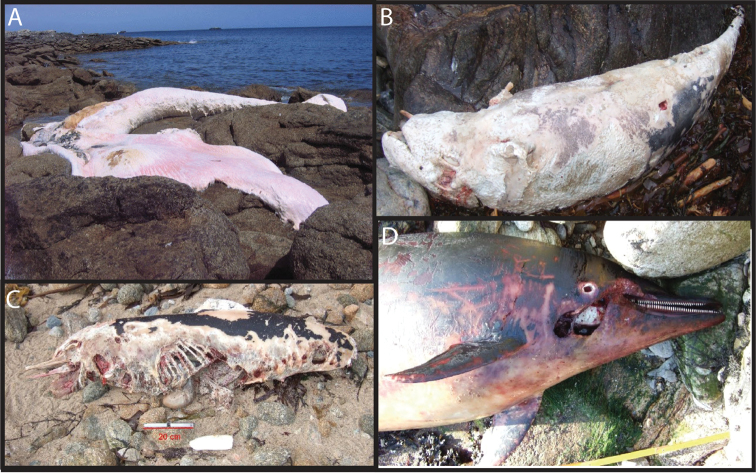
Examples of marine mammals stranded along the coasts of Brittany and the species-level identifications of which were determined or confirmed thanks to DNA barcoding. **A** Sample Ms250511, stranded on the “Île de Sein” during May 2011, and identified as a *Balaenoptera physalus* **B** Sample Ds160111, stranded on the Ushant Island during January 2011, and identified as a *Grampus griseus* **C** Sample Ds130211, stranded on the Ushant Island in February 2011, and identified as belonging to the Delphininae subfamily (putatively identified as a *Delphinus delphis* on the nMDS plot in Figure 5) **D** Sample Ds080410 stranded on the Ushant Island during April 2010, and identified as belonging to the Delphininae (putatively identified as a *Stenella coeruleoalba* on the nMDS plot in Figure 5).

We then determined COI sequences from 10 cetacean samples whose species could not be determined accurately using morphological characters (Figure 4), either because only parts of the animal were recovered (Figure 4A) or because of the highly degraded state of the carcasses (Figure 4C). COI sequences of good qualities were obtained from all these samples, and three of them were identified unambiguously using the BOLD identification engine: Ms250511 was identified as a *Balaenoptera physalus*, Ds160111 as a *Grampus griseus* and Ds290811 as a *Phocoena phocoena*. The other seven samples were Delphininae, as confirmed by COI sequences. Yet, neither the BOLD identification engine, nor a BLAST search on GenBank allowed a more precise determination. We therefore sequenced MCR, which is more variable than COI, from six unidentified samples. BLAST searches on GenBank confirmed the COI results: all these samples were Delphininae, but a more precise identification could not be achieved.

We constructed a nMDS plot of the distances between MCR sequences of *Stenella coeruleoalba*, *Stenella frontalis* and *Delphinus delphis* taken from GenBank: for *Stenella coeruleoalba*, we used sequences AM498725, AM498723, AM498721, AM498719, AM498717, AM498715, AM498713, AM498711, AM498709, AM498707 (Mace et al. unpublished), for *Delphinus delphis* FM211560, FM211553, FM211545, FM211535, FM211527, FM211519, FM211511, FM211503, FM211495 (Mirimin et al. 2009) and DQ520121, DQ520117, DQ520113, DQ520109, DQ520105 (Hildebrandt et al. unpublished) and for *Stenella frontalis* GQ5041986, GQ5041987, GQ5041988, GQ5041989, GQ5041990, GQ5041991, GQ5041992, GQ5041993, GQ5041994, GQ5041995 (Kingston et al. 2009).

**Figure 5.**
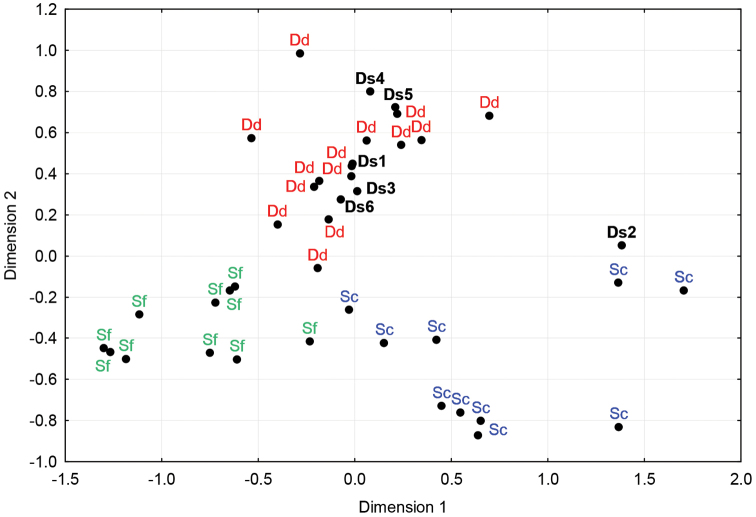
Non-metric Multidimensional Scaling plot of K2P-distance between MCR sequences of *Stenella coeruleoalba* (in blue), *Delphinus delphis* (in red) and *Stenella frontalis* (in green). Individuals of each species are clearly clustered together, and unidentified samples (in black) stranded along the coasts of Brittany group with one of the three species. Dd280211A (Ds1), Ds130210 (Ds3), Ds230409 (Ds4), Ds250412 (Ds5) and Sc210910 (Ds6) are putatively identified as *Delphinus delphis*, whereas Ds080410 (Ds2) would more likely belong to *Stenella coeruloalba*.

The three species were clearly discriminated by the nMDS (Figure 5). The posterior probabilities are given in Appendix 2. This analysis suggests that five of our unidentified samples could belong to *Delphinus delphis*, and one to *Stenella coeruleoalba*.

### Intraspecific variation of COI and MCR in harbour porpoise and grey seal

For the intraspecific analysis of the harbour porpoise, we included 17 additional samples of animals stranded or by-caught from the Bay of Biscay (Appendix 1, Alfonsi et al. 2012). All in all, we compared 35 sequences of grey seals, and 45 of harbour porpoises. As expected, MCR sequences were more polymorphic than COI: in harbour porpoise, 3.8% of the MCR positions were polymorphic vs. 1.30% in COI, while 4.73% of the MCR positions in the grey seal were polymorphic vs. 0.75% in COI (Table 4). Hence, MCR was 3× more polymorphic than COI in harbour porpoise and 6x in grey seals. Haplotype and nucleotide diversities were also higher for MCR than for COI.

**Table 4.** Comparison of intraspecific COI and MCR polymorphisms for grey seal and harbour porpoise

	Harbour porpoise (*Phocoena phocoena*)		Grey seal (*Halichoerus grypus*)	
Markers	**COI**	**MCR**	**COI**	**MCR**
Number of sequences	45	45	35	35
Sequence length (bp)	610	579	658	482
Haplotypes	9	14	6	14
Polymorphic sites	8	22	5	23
Polymorphism	1.30%	3.80%	0.76%	4.77%
Haplotype diversity	0.695	0.832	0.553	0.935
Nucleotide diversity	0.00242	0.00632	0.00098	0.00945

**Figure 6.**
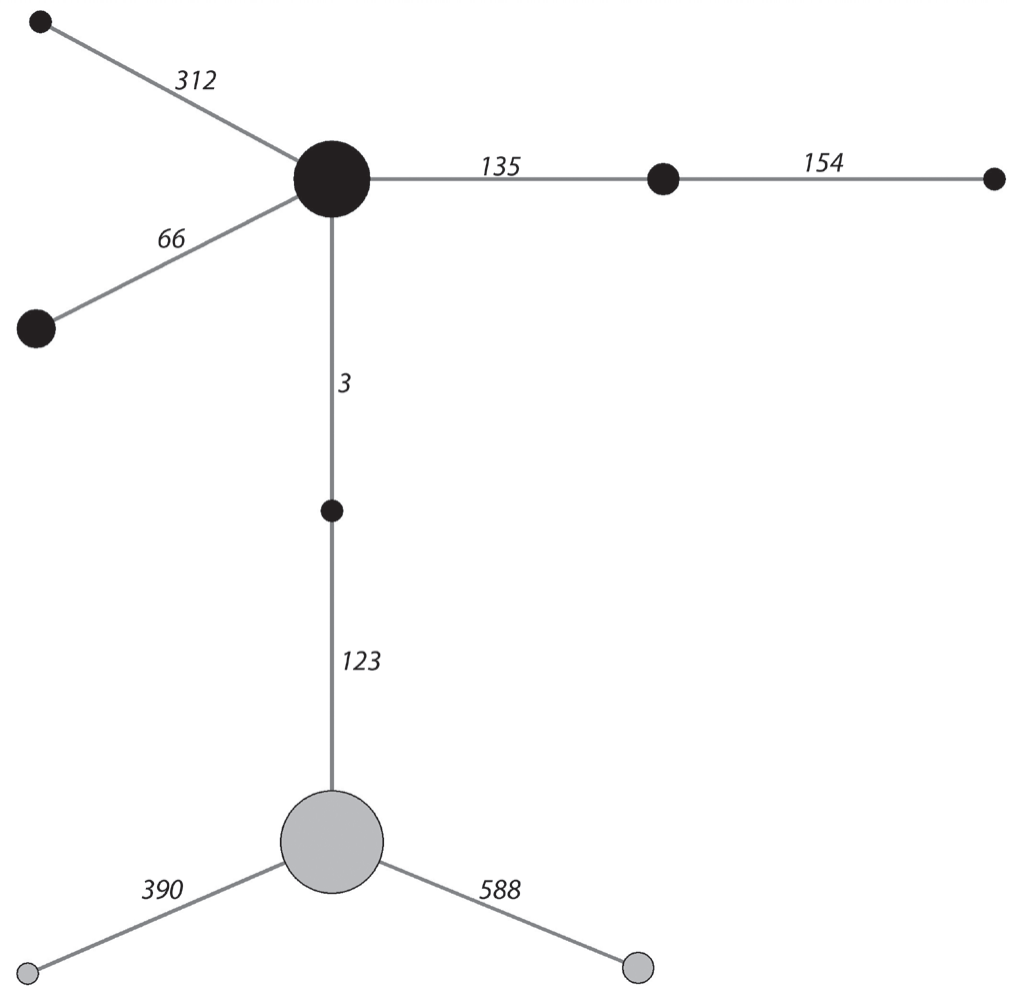
Haplotype network established from the COI sequences of 45 harbour porpoises stranded along the Atlantic coast of France (Appendix 1). Numbers on a line connecting two haplotypes correspond to the sequence position of the mutation differentiating these haplotypes. Two mitochondrial haplogroups appear (black circles - grey circles), that group the same individuals as the haplogroups alpha and beta determined using MCR polymorphisms and described in Alfonsi et al. (2012).

The haplotype network of the COI sequences in harbour porpoises clearly differentiated two haplogroups (Figure 6), that correspond perfectly to those described for MCR in Alfonsi et al. (2012).

## Discussion

Stranding networks collect opportunistic data that are ecologically significant (Borsa 2006, Jung et al. 2009, Peltier et al. 2013), although, among other parameters, data quality control may deserve a special attention (Evans and Hammond 2004). Stranding networks can also collect skin and muscle samples that can be used for genetic analysis, therefore contributing to the construction of biological sample banks which are of high value when working with marine mammals.

The aim of this study was to evaluate the feasibility of a routine use of DNA barcoding in a stranding network; and to determine which gains this use could bring in terms of data relevance. The Brittany stranding network is a part of the French stranding network, and has to analyze an average of around 150 marine mammal strandings per year, with a high species biodiversity (19 species during 2003–2012).

### Can COI be used as an appropriate species identification tool for marine mammals in the frame of a stranding network?

We obtained DNA sequences of good quality for almost all the samples studied, whatever their origin, their collectors, or even their state of degradation. This is consistent with the numerous molecular genetic studies that have used samples taken on stranded cetaceans or pinnipeds (e.g. Gaspari et al. 2006, Amaral et al. 2007, Fontaine et al. 2007, Mirimin et al. 2009,^2011^, Alfonsi et al. 2012).

Viricel and Rosel (2012) previously demonstrated that COI sequences allowed identifying cetacean species, except for a few closely related Delphinidae species (see also Amaral et al. 2007). As expected, our NJ tree matched the overall classification, and the distance-based analysis identified correctly the sequences to the species levels for all cetaceans except within the Delphininae. The three species of pinnipeds analyzed were also unambiguously distinguished on the basis of their COI sequences.

The quality of the whole functioning and organization of the stranding network, from the field-work achieved by the correspondents to the preservation of the samples is therefore confirmed by our study. All the samples analyzed by DNA barcoding led to correct identification of the expected species with no exceptions.

We obtained COI good quality sequences for 10 unidentified animals, some of which were in a highly degraded body state. This showed that DNA barcoding can help to identify such specimens, which represent more than 16% of the stranded animals in the period 2003–2012. Hence, a routine use of DNA barcoding would noticeably decrease the proportion of unidentified animals.

### The case of the Delphininae

Within the Delphininae, species are difficult to discriminate (Amaral et al. 2007, 2012, Viricel and Rosel 2012). In particular, *Delphinus delphis*, *Stenella coeruleoalba* and *Stenella frontalis* show very low interspecific COI distances, which do not allow distinguishing the species accurately. Other mitochondrial loci, such as MCR and cyt *b*, are neither very effective in this matter (Amaral et al. 2007, Viricel and Rosel 2012). This is attributed to recent and rapid radiation events in the subfamily, and it leads to problematic results in molecular taxonomic studies (Kingston et al. 2009, Amaral et al. 2012, Viricel and Rosel 2012, Perrin 2013). In our case, these three species produced COI and MCR sequences that did not allow to associate samples with species names, neither with the identification engine on BOLD, nor with a distance tree or a BLAST search on GenBank. nMDS of genetic distances is known to uncover sample clustering (e.g. Geffen et al. 2004, Maltagliati et al. 2006, Alfonsi et al. 2012, Weckworth et al. 2012). As such, nMDS clustering of MCR sequence distances of *Delphinus delphis*, *Stenella coeruleoalba* and *Stenella frontalis*, chosen randomly on GenBank among Atlantic samples, showed that individuals of the three species formed separate groups. Moreover, each individual had a high posterior probability to belong to the right group, except for one sample (i.e. 97.0% of the assignments were successful), so that all our unidentified samples could be putatively identified to the species level, based on the nMDS plot and its posterior probabilities.

### Can DNA barcoding increase the accuracy of the data listed by the stranding network?

DNA barcoding is informative for animals that belong to species that infrequently strand along the coasts of Brittany, which can involve either species living far off the coasts or living in deep water, but also exotic species. Such species can be more difficult to identify by the field correspondents simply because of their scarcity. Along the coast of Brittany, we observed a *Stenella frontalis*, a temperate to tropical Atlantic Ocean inhabitant, and three species of arctic seals (*Phoca hispida*, *Cystophora cristata* and *Phoca groenlandica*). It is likely that other members of such rare species are listed among the “undetermined” species, just because their morphological characteristics are less well known by field correspondents. Additionally, a species that rarely strands along the French coast may be mistakenly identified as its more common sister-species. This issue can be illustrated by the case of the two pilot whale species: *Globicephala melas*, the long-finned pilot whale, commonly strands along the French Atlantic coast, while only a few stranding events of *Globicephala macrorhynchus*, the short-finned pilot whale, have been reported (the Bay of Biscay is the northern limit of the geographical range of *Globicephala macrorhynchus*). The two species have overlaping morphological characters, which adds to the difficulty of detecting rare stranding events of *Globicephala macrorhynchus* based on morphological data only (Viricel and Sabatier unpublished data). A systematic use of DNA barcoding when morphological taxonomic characteristics are not straightforward, would clearly lower the percentage of exotic animals not listed. The existence of natural interspecific hybrids between the two *Globicephala* sister-species (Miralles et al. 2013), as between other cetacean species (e.g. Bérubé and Aguilar 1998, Willis et al. 2004) still reinforces the interest of such a monitoring based on molecular data.

It is important to note that a main limitation of DNA barcoding is the use of a single locus, leading to some problematic species identification such as within the Delphininae, but also to an inability to detect hybrids without complementary genetic studies. This limitation may well be removed in the near future thanks to next-generation sequencing, allowing the accumulation of large amount of DNA sequence data in a cost-effective manner. Multi-locus barcoding, including mitochondrial and nuclear polymorphic loci, will certainly represent a next step for the barcoding community.

A routine use of DNA barcoding could also allow monitoring the marine mammal biodiversity at intraspecific levels. For instance, global climate change has some effects on genetic diversity that must be studied and quantified (Pauls et al. 2012), in particular in the marine realm. Knowledge of the existence of distinct genetic groups or populations, of the history of their formation and of their movements are of a first importance to ecological understandings of natural populations, and also to the conservation efforts dedicated to them. Around the coast of Brittany, different species of marine mammals have shown variations in abundance in the last decades (Vincent et al. 2005, Jung et al. 2009). Using samples from the French Stranding Network and MCR polymorphisms, we have recently shown that two previously separated, genetically distinct, populations of harbour porpoises are now admixing along the Atlantic coast of France (Alfonsi et al. 2012). These results were unexpected according to previous work (Tolley and Rosel 2006, Fontaine et al. 2007). In this study, we show that this genetic clustering would also have been detected using COI polymorphisms, thus reinforcing the interest of a routine use of DNA barcoding in conjunction with the stranding network.

### Contributions of our study to the Barcoding of Life Database

This project is part of the collaboration between the Laboratory BioGeMME of the “Université de Bretagne Occidentale” (Brest, France), Océanopolis, a public private company (http://www.oceanopolis.com), the “Parc naturel marin d’Iroise” (http://www.parc-marin-iroise.com) and the French Stranding Network, coordinated by *Pelagis*, Université de La Rochelle, France. All the specimens and sequence data described in this manuscript are deposited in BOLD under the institution called “Oceanopolis-BioGeMME” in two projects, UMMB and IMMB. Our mixed institution became the first contributor to BOLD for the Cetacea, as well as for the Phocidae, and these two BOLD projects will be publicly available, and all the sequences published on GenBank.

## Appendix 1

List of the 46 *Phocoena phocoena* analyzed. (doi: 10.3897/zookeys.365.5873.app1) File format: Microsoft Word file (doc).

## Appendix 2

Posterior probabilities for species identification determined by the nMDS analysis. (doi: 10.3897/zookeys.365.5873.app2) File format: Microsoft Word file (doc).

## References

[B1] Alfonsi E Hassani S Carpentier F-G Le Clec’h J-Y Dabin W Van Canneyt O Fontaine M C Jung J-L (2012) A European Melting Pot of Harbour Porpoise in the French Atlantic Coasts Inferred from Mitochondrial and Nuclear Data. PLoS ONE 7: e44425. doi: 10.1371/journal.pone.0044425 22984507 PMC3440431

[B2] Amaral AR Jackson JA Möller LM Beheregaray LB Coelho MM (2012) Species tree of a recent radiation: The subfamily Delphininae (Cetacea, Mammalia). Molecular Phylogenetics and Evolution 64: 243-253. doi: 10.1016/j.ympev.2012.04.004 22503758

[B3] Amaral AR Sequeira M Coelho MM (2007) A first approach to the usefulness of cytochrome c oxidase I barcodes in the identification of closely related delphinid cetacean species. Marine and Freshwater Research 58: 505-510. doi: 10.1071/MF07050

[B4] Ballard JWO Whitlock MC (2004) The incomplete natural history of mitochondria. Molecular Ecology 13: 729-744. doi: 10.1046/j.1365-294X.2003.02063.x 15012752

[B5] Benson DA Karsch-Mizrachi I Lipman DJ Ostell J Sayers EW (2010) GenBank. Nucleic Acids Research 39 (Database), D32–D37. doi: 10.1093/nar/gkp1024 21071399 PMC3013681

[B6] Bérubé M Aguilar A (1998) A new hybrid between a blue whale *Balaenoptera musculus* and a fin whale *B. physalus*: frequency and implications of hybridization. Marine Mammal Science 14: 82-98. doi: 10.1111/j.1748-7692.1998.tb00692.x

[B7] Borisenko AV Lim BK Ivanova NV Hanner RH Hebert PDN (2008) DNA barcoding in surveys of small mammal communities: a field study in Suriname. Molecular Ecology Resources 8: 471-479. doi: 10.1111/j.1471-8286.2007.01998.x 21585824

[B8] Borsa P (2006) Marine mammal strandings in the New Caledonia region, Southwest Pacific. Comptes Rendus Biologies 329: 277-288. doi: 10.1016/j.crvi.2006.01.004 16644500

[B9] Clare EL Lim BK Engstrom MD Eger JL Hebert PDN (2007) DNA barcoding of Neotropical bats: species identification and discovery within Guyana. Molecular Ecology Notes 7: 184–190. doi: 10.1111/j.1471-8286.2006.01657.x

[B10] Dawnay N Ogden R Mcewing R Carvalho G Thorpe R (2007) Validation of the barcoding gene COI for use in forensic genetic species identification. Forensic Science International 173: 1-6. doi: 10.1016/j.forsciint.2006.09.013 17300895

[B11] Evans P Hammond P (2004) Monitoring cetaceans in European waters. Mammal Review 34: 131–156. doi: 10.1046/j.0305-1838.2003.00027.x

[B12] Felsenstein J (1989) PHYLIP – Phylogeny Inference Package (Version 3.2). Cladistics 5: 164-166.

[B13] Fontaine MC Baird SJ Piry S Ray N Tolley KA Duke S Birkun A Ferreira M Jauniaux T Llavona Á Öztürk BA Öztürk A Ridoux V Rogan E Sequeira M Siebert U Vikingsson GA Bouquegneau J-M Michaux JR (2007) Rise of oceanographic barriers in continuous populations of a cetacean: the genetic structure of harbour porpoises in Old World waters. BMC Biology 5: 30. doi: 10.1186/1741-7007-5-30 17651495 PMC1971045

[B14] Gaspari S Airoldi S Hoelzel AR (2006) Risso’s dolphins (*Grampus griseus*) in UK waters are differentiated from a population in the Mediterranean Sea and genetically less diverse. Conservation Genetics 8: 727-732. doi: 10.1007/s10592-006-9205-y

[B15] Geffen E Anderson MJ Wayne RK (2004) Climate and habitat barriers to dispersal in the highly mobile grey wolf. Molecular Ecology 13: 2481-2490. doi: 10.1111/j.1365-294X.2004.02244.x 15245420

[B16] Hajibabaei M Singer GA Clare EL Hebert PDN (2007) Design and applicability of DNA arrays and DNA barcodes in biodiversity monitoring. BMC Biology 5: 24. doi: 10.1186/1741-7007-5-24 17567898 PMC1906742

[B17] Hall TA (1999) BioEdit: a user-friendly biological sequence alignment editor and analysis program for Windows 95/98/NT. Nucleic Acids Symposium Series 41: 95-98.

[B18] Hartl DL Clark AG (2007) Principles of Population Genetics. Sinauer and Associates, Sunderland, MA.

[B19] Hebert PDN Cywinska A Ball SL deWaard JR (2003) Biological identifications through DNA barcodes. Proceedings of the Royal Society of London B 270: 313-321. doi: 10.1098/rspb.2002.2218 PMC1691236 12614582

[B20] Hebert PDN Stoeckle MY Zemlak TS Francis CM (2004) Identification of Birds through DNA Barcodes. PLoS Biology 2: e312. doi: 10.1371/journal.pbio.0020312 15455034 PMC518999

[B21] Jung J-L Stéphan E Louis M Alfonsi E Liret C Carpentier F-G Hassani S (2009) Harbour porpoises (*Phocoena phocoena*) in north-western France: aerial survey, opportunistic sightings and strandings monitoring. Journal of the Marine Biological Association of the United Kingdom 89: 1045-1050. doi: 10.1017/S0025315409000307

[B22] Kimura M (1980) A simple method for estimating evolutionary rate of base substitutions through comparative studies of nucleotide sequences. Journal of Molecular Evolution 16: 111–120. doi: 10.1007/BF01731581 7463489

[B23] Kingston SE Adams LD Rosel PE (2009) Testing mitochondrial sequences and anonymous nuclear markers for phylogeny reconstruction in a rapidly radiating group: molecular systematics of the Delphininae (Cetacea: Odontoceti: Delphinidae). BMC Evolutionary Biology 9: 245. doi: 10.1186/1471-2148-9-245 PMC2770059 19811651

[B24] Kmiec B Woloszynska M Janska H (2006) Heteroplasmy as a common state of mitochondrial genetic information in plants and animals. Current Genetics 50: 149-159. doi: 10.1007/s00294-006-0082-1 16763846

[B25] Lambert DM (2005) Is a Large-Scale DNA-Based Inventory of Ancient Life Possible? Journal of Heredity 96: 279–284. doi: 10.1093/jhered/esi035 15731217

[B26] LeDuc RG Perrin WF Dizon AE (1999) Phylogenetic relationships among the delphinid cetaceans based on full cytochrome b sequences. Marine Mammal Science 15: 619-648. doi: 10.1111/j.1748-7692.1999.tb00833.x

[B27] Librado P Rozas J (2009) DnaSP v5: a software for comprehensive analysis of DNA polymorphism data. Bioinformatics 25: 1451-1452. doi: 10.1093/bioinformatics/btp187 19346325

[B28] Maltagliati F Lai T Casu M Valdesalici S Castelli A (2006) Identification of endangered Mediterranean cyprinodontiform fish by means of DNA inter-simple sequence repeats (ISSRs). Biochemical Systematics and Ecology 34: 626-634. doi: 10.1016/j.bse.2006.02.003

[B29] McGowen MR (2011) Toward the resolution of an explosive radiation—A multilocus phylogeny of oceanic dolphins (Delphinidae). Molecular Phylogenetics and Evolution 60: 345–357. doi: 10.1016/j.ympev.2011.05.003 21600295

[B30] Miralles L Lens S Rodriguez-Folgar A Carrillo M Martin V Mikkelsen B Garcia-Vazquez E (2013) Interspecific Introgression in Cetaceans: DNA Markers Reveal Post-F1 Status of a Pilot Whale. PLoS ONE 8: e69511. doi: 10.1371/journal.pone.0069511 23990883 PMC3747178

[B31] Mirimin L Westgate A Rogan E Rosel P Andrew R Coughlan J Cross T (2009) Population structure of short-beaked common dolphins (*Delphinus delphis*) in the North Atlantic Ocean as revealed by mitochondrial and nuclear genetic markers. Marine Biology 156: 821–834. doi: 10.1007/s00227-009-1147-8

[B32] Mirimin L Miller R Dillane E Berrow SD Ingram S Cross T Rogan E (2011) Fine-scale population genetic structuring of bottlenose dolphins in Irish coastal waters. Animal Conservation 14: 342-353. doi: 10.1111/j.1469-1795.2010.00432.x

[B33] Pauls SU Nowak C Bálint M Pfenninger M (2012) The impact of global climate change on genetic diversity within populations and species. Molecular Ecology 22: 925-946. doi: 10.1111/mec.12152 23279006

[B34] Peltier H Baagøe HJ Camphuysen KCJ Czeck R Dabin W Daniel P Deaville R Haelters J Jauniaux T Jensen LF Jepson PD Keijl GO Siebert U Van Canneyt O Ridoux V (2013) The Stranding Anomaly as Population Indicator: The Case of Harbour Porpoise *Phocoena phocoena* in North-Western Europe. PLoS ONE 8: e62180. doi: 10.1371/journal.pone.0062180 23614031 PMC3632559

[B35] Perrin WF Rosel PE Cipriano F (2013) How to contend with paraphyly in the taxonomy of the delphinine cetaceans? Marine Mammal Science. doi: 10.1111/mms.12051

[B36] Ratnasingham S Hebert PD (2007) BOLD: The Barcode of Life Data System (www.barcodinglife.org). Molecular Ecology Notes 7: 355-364. doi: 10.1111/j.1471-8286.2007.01678.x 18784790 PMC1890991

[B37] Shokralla S Singer G Hajibabaei M (2010) Direct PCR amplification and sequencing of specimens’ DNA from preservative ethanol. BioTechniques 48: 233-234. doi: 10.2144/000113362 20359306

[B38] Tamura K Peterson D Peterson N Stecher G Nei M Kumar S (2011) MEGA5: Molecular Evolutionary Genetics Analysis Using Maximum Likelihood, Evolutionary Distance, and Maximum Parsimony Methods. Molecular Biology and Evolution 28: 2731-2739. doi: 10.1093/molbev/msr121 21546353 PMC3203626

[B39] Thompson JD Higgins DG Gibson TJ (1994) CLUSTAL W: improving the sensitivity of progressive multiple sequence alignment through sequence weighting, position- specific gap penalties and weight matrix choice. Nucleic Acids Research 22: 4673-4680. doi: 10.1093/nar/22.22.4673 7984417 PMC308517

[B40] Thompson K Baker CS Van Helden A Patel S Millar C Constantine R (2012) The world’s rarest whale. Current Biology 22, R905–R906. doi: 10.1016/j.cub.2012.08.055 23137682

[B41] Toews DPL Brelsford A (2012) The Biogeography of Mitochondrial and Nuclear Discordance in Animals. Molecular Ecology 21: 3907-3930. doi: 10.1111/j.1365-294X.2012.05664.x 22738314

[B42] Tolley KA Rosel PE (2006) Population structure and historical demography of eastern North Atlantic harbour porpoises inferred through mtDNA sequences. Marine Ecology Progress Series 327: 297-308. doi: 10.3354/meps327297

[B43] Valentini A Pompanon F Taberlet P (2009) DNA barcoding for ecologists. Trends in Ecology & Evolution 24: 110-117. doi: 10.1016/j.tree.2008.09.011 19100655

[B44] Vincent C Fedak M Mcconnell B Meynier L Saintjean C Ridoux V (2005) Status and conservation of the grey seal, *Halichœrus grypus*, in France. Biological Conservation 126: 62–73. doi: 10.1016/j.biocon.2005.04.022

[B45] Viricel A Rosel PE (2012) Evaluating the utility of *cox1* for cetacean species identification. Marine Mammal Science 28: 37-62. doi: 10.1111/j.1748-7692.2010.00460.x

[B46] Vollmer NL Viricel A Wilcox L Moore MK Rosel P (2011) The occurrence of mtDNA heteroplasmy in multiple cetacean species. Current Genetics 57: 115-131. doi: 10.1007/s00294-010-0331-1 21234756

[B47] Ward RD Hanner R Hebert PDN (2009) The campaign to DNA barcode all fishes, FISH-BOL. Journal of Fish Biology 74: 329-356. doi: 10.1111/j.1095-8649.2008.02080.x 20735564

[B48] Weckworth BV Musiani M Mcdevitt AD Hebblewhite M Mariani S (2012) Reconstruction of caribou evolutionary history in Western North America and its implications for conservation. Molecular Ecology 21: 3610-3624. doi: 10.1111/j.1365-294X.2012.05621.x 22612518

[B49] Wiemers M Fiedler K (2007) Does the DNA barcoding gap exist? – a case study in blue butterflies (Lepidoptera: Lycaenidae). Frontiers in Zoology 4: 8. doi: 10.1186/1742-9994-4-8 17343734 PMC1838910

[B50] Willis PM Crespi BJ Dill LM Baird RW Bradley Hanson M (2004) Natural hybridization between Dall’s porpoises (*Phocoenoides dalli*) and harbour porpoises (*Phocoena phocoena*). Canadian Journal of Zoology 82: 828-834. doi: 10.1139/z04-059

