# Supplementary material for: The use of DNA barcoding to monitor the marine mammal biodiversity along the French Atlantic coast
Source: Zookeys. 2013 Dec 30;(365):5–24. doi: 10.3897/zookeys.365.5873 (PMC3890668; doi:10.3897/zookeys.365.5873)
Supplement: Supplementary file 1 — List of the 46 Phocoena phocoena analyzed. (doi: 10.3897/zookeys.365.5873.app1) File format: Microsoft Word file (doc). [file ZooKeys-365-005-s001.doc]

|  | **Reference on BOLD** | **Reference in**  **Alfonsi et al. (2012)** | **Place of stranding** | **Departments (Territorial**  **and Administrative Division**  **in France)** |
| --- | --- | --- | --- | --- |
| 1 | pp240100 | ppbec1 | Brittany | 29 |
| 2 | pp140904 | ppbec2 | Brittany | 29 |
| 3 | pp111204 | ppbec3 | Brittany | 29 |
| 4 | pp011005 | ppbec7 | Brittany | 29 |
| 5 | pp210306 | ppbec8 | Brittany | 22 |
| 6 | pp090506 | ppbec9 | Brittany | 29 |
| 7 | pp051006 | ppbec10 | Brittany | 29 |
| 8 | pp111006 | ppbec11 | Brittany | 29 |
| 9 | pp190107 | ppbec12 | Brittany | 56 |
| 10 | pp260207 | ppbec13 | Brittany | 29 |
| 11 | pp240907 | ppbec14 | Brittany | 29 |
| 12 | pp270907 | ppbec15 | Brittany | 29 |
| 13 | pp071207 | ppbec17 | Brittany | 29 |
| 14 | pp070208 | ppbec18 | Brittany | 56 |
| 15 | pp120908 | ppbec23 | Brittany | 29 |
| 16 | pp150908 | ppbec24 | Brittany | 29 |
| 17 | pp061008 | ppbec25 | Brittany | 29 |
| 18 | pp160808 | ppbec21 | Brittany | 29 |
| 19 | pp010508 | ppbec19 | Brittany | 29 |
| 20 | pp031107 | ppbec16 | Brittany | 29 |
| 21 | pp261208 | ppbec26 | Brittany | 29 |
| 22 | pp070908 | ppbec22 | Brittany | 29 |
| 23 | pp240708 | ppbec20 | Brittany | 22 |
| 24 | pp150909 | ppbec29 | Brittany | 29 |
| 25 | pp100909 | ppbec28 | Brittany | 29 |
| 26 | pp040609 | ppbec27 | Brittany | 29 |
| 27 | pp080410 | ppbec31 | Britanny | 29 |
| 28 | pp190310 | ppbec30 | Britanny | 29 |
| 29 | pp190111 | -* | Britanny | 29 |
| 30 | - | ppbob1 | Bay of Biscay | 33 |
| 31 | - | ppbob2 | Bay of Biscay | 85 |
| 32 | - | ppbob4 | Bay of Biscay | 33 |
| 33 | - | ppbob5 | Bay of Biscay | 33 |
| 34 | - | ppbob6 | Bay of Biscay | 33 |
| 35 | - | ppbob8 | Bay of Biscay | 33 |
| 36 | - | ppbob11 | Bay of Biscay | 33 |
| 37 | - | ppbec4 | Brittany | 56 |
| 38 | - | ppbec5 | English Channel | 76 |
| 39 | - | ppbec6 | English Channel | 62 |
| 40 | - | ppbob12 | Bay of Biscay | 33 |
| 41 | - | ppbob13 | Bay of Biscay | 33 |
| 42 | - | ppbob14 | Bay of Biscay | 33 |
| 43 | - | ppbob15 | Bay of Biscay | 33 |
| 44 | - | ppbob19 | Bay of Biscay | 40 |
| 45 | - | ppbob16 | Bay of Biscay | 33 |
| 46 | - | ppbob18 | Bay of Biscay | 33 |

Table S1: List of the 46 *Phocoena phocoena* analyzed. References of each sample on the BOLD project IMMB are given for the 29 first samples (the other ones do not belong to the BOLD project). Correspondence with the references used in Alfonsi et al. 2012 for the same individuals are specified.

(*): pp190111 was not included in Alfonsi et al. (2012).

Territorial and Administrative Division in France ("départements"): 29 (Finistère), 33 (Gironde), 40 (Landes), 56 (Morbihan) 62 (Pas de calais), 76 (Seine-Maritime)
