# Supplementary material for: The use of DNA barcoding to monitor the marine mammal biodiversity along the French Atlantic coast
Source: Zookeys. 2013 Dec 30;(365):5–24. doi: 10.3897/zookeys.365.5873 (PMC3890668; doi:10.3897/zookeys.365.5873)
Supplement: Supplementary file 2 — Posterior probabilities for species identification determined by the nMDS analysis. (doi: 10.3897/zookeys.365.5873.app2) File format: Microsoft Word file (doc). [file ZooKeys-365-005-s002.doc]

|  | **Posterior probability of belonging to** | | |  |
| --- | --- | --- | --- | --- |
| **Species-Genbank Reference** | **Sc** | **Dd** | **Sf** | **Determined species** |
| Sc-AM498725.1 | 1.000000 | 0.000000 | 0.000000 | *Stenella coeruleoalba* |
| Sc-AM498723.1 | 0.999954 | 0.000000 | 0.000046 | *Stenella coeruleoalba* |
| Sc-AM498721.1 | 0.999994 | 0.000005 | 0.000001 | *Stenella coeruleoalba* |
| Sc-AM498719.1 | 0.989320 | 0.000349 | 0.010330 | *Stenella coeruleoalba* |
| Sc-AM498717.1 | 0.999940 | 0.000000 | 0.000060 | *Stenella coeruleoalba* |
| Sc-AM498715.1 | 0.999974 | 0.000000 | 0.000026 | *Stenella coeruleoalba* |
| Sc-AM498713.1 | 0.900884 | 0.001148 | 0.097968 | *Stenella coeruleoalba* |
| Sc-AM498711.1 | 0.999698 | 0.000000 | 0.000302 | *Stenella coeruleoalba* |
| Sc-AM498709.1 | 0.999996 | 0.000004 | 0.000000 | *Stenella coeruleoalba* |
| **Sc-AM498707.1** | **0.254159** | **0.043760** | **0.702081** | ***Stenella frontalis*** |
| Dd-FM211560.1 | 0.000522 | 0.904135 | 0.095342 | *Delphinus delphis* |
| Dd-FM211553.1 | 0.000002 | 0.999990 | 0.000008 | *Delphinus delphis* |
| Dd-FM211545.1 | 0.000002 | 0.998616 | 0.001383 | *Delphinus delphis* |
| Dd-FM211535.1 | 0.000892 | 0.664208 | 0.334900 | *Delphinus delphis* |
| Dd-FM211527.1 | 0.000000 | 0.999382 | 0.000618 | *Delphinus delphis* |
| Dd-FM211519.1 | 0.000015 | 0.999776 | 0.000210 | *Delphinus delphis* |
| Dd-FM211511.1 | 0.000000 | 0.999822 | 0.000178 | *Delphinus delphis* |
| Dd-FM211503.1 | 0.000000 | 0.999999 | 0.000001 | *Delphinus delphis* |
| Dd-FM211495.1 | 0.000003 | 0.947209 | 0.052788 | *Delphinus delphis* |
| Dd-DQ520121.1 | 0.000000 | 0.999967 | 0.000032 | *Delphinus delphis* |
| Dd-DQ520117.1 | 0.000001 | 0.995285 | 0.004713 | *Delphinus delphis* |
| Dd-DQ520113.1 | 0.000010 | 0.996175 | 0.003815 | *Delphinus delphis* |
| Dd-DQ520109.1 | 0.000002 | 0.991619 | 0.008378 | *Delphinus delphis* |
| Dd-DQ520105.1 | 0.000003 | 0.999757 | 0.000240 | *Delphinus delphis* |
| Sf-GQ504195.1 | 0.000141 | 0.074688 | 0.925171 | *Stenella frontalis* |
| Sf-GQ504194.1 | 0.000006 | 0.000102 | 0.999893 | *Stenella frontalis* |
| Sf-GQ504193.1 | 0.000082 | 0.067209 | 0.932709 | *Stenella frontalis* |
| Sf-GQ504192.1 | 0.000008 | 0.000103 | 0.999889 | *Stenella frontalis* |
| Sf-GQ504191.1 | 0.000184 | 0.001489 | 0.998327 | *Stenella frontalis* |
| Sf-GQ504190.1 | 0.110536 | 0.004258 | 0.885205 | *Stenella frontalis* |
| Sf-GQ504189.1 | 0.000049 | 0.028326 | 0.971625 | *Stenella frontalis* |
| Sf-GQ504188.1 | 0.000009 | 0.001694 | 0.998298 | *Stenella frontalis* |
| Sf-GQ504187.1 | 0.000039 | 0.000063 | 0.999899 | *Stenella frontalis* |
| Sf-GQ504186.1 | 0.003433 | 0.000728 | 0.995839 | *Stenella frontalis* |
| **BOLD reference (our samples)** | | | |  |
| **Dd280211A** | 0.000002 | **0.998405** | 0.001593 | ***Delphinus delphis*** |
| **Ds080410** | **0.998108** | 0.001881 | 0.000011 | ***Stenella coeruleoalba*** |
| **Ds130210** | 0.000026 | **0.993354** | 0.006620 | ***Delphinus delphis*** |
| **Ds230409** | 0.000000 | **0.999993** | 0.000007 | ***Delphinus delphis*** |
| **Ds250412** | 0.000000 | **0.999982** | 0.000018 | ***Delphinus delphis*** |
| **Sc210910** | 0.000031 | **0.986569** | 0.013400 | ***Delphinus delphis*** |

Table S2: Posterior probabilities for species identification determined by the nMDS analysis. Only one sample (AM-498707.1), identified on genbank as a *Stenella coeruleoalba*, is putatively misidentified by our analysis as a *S. frontalis*
